# Fetal Intervention for Refractory Supraventricular Tachycardia Complicated by Hydrops Fetalis

**DOI:** 10.1155/2022/5148250

**Published:** 2022-03-12

**Authors:** Jessian L. Munoz, Ariana L. Lewis, Jun Song, Patrick S. Ramsey

**Affiliations:** ^1^University of Texas Health Science Center at San Antonio/University Health System, Department of Obstetrics & Gynecology-Division of Maternal Fetal Medicine, San Antonio, Texas, USA; ^2^University of Texas Health Science Center at San Antonio/University Health System, Department of Obstetrics & Gynecology, San Antonio, Texas, USA

## Abstract

*Introduction*. Few reports have shown promising treatments for refractory fetal tachycardia. Data are limited regarding optimal treatment, route of treatment, and medication dosages. Over 90% of cases of fetal tachycardia can be attributed to supraventricular tachycardia (SVT). The first-line treatment of fetal SVT is transplacental digoxin. *Case Presentation*. We present the management of a patient with fetal tachyarrhythmia diagnosed at 24 weeks and offer a unique approach for treatment. Fetal intramuscular injection of 72.3 mcg of digoxin allowed for resolution of SVT and sustained normal sinus rhythm. Further assessment in the third trimester showed persistent hydrops in the setting of mirror (Ballantyne's) syndrome resulting in delivery. *Discussion/Conclusion*. Our observations suggest that a one-time injection of digoxin allows for complete resolution of SVT. Utilizing an invasive approach for management of SVT that is resistant to traditional treatment modalities appears to both be therapeutic and decrease maternal adverse effects associated with more toxic effects of other transplacental medications.

## 1. Introduction

Invasive approaches to treatment of fetal anomalies have dramatically changed the landscape of fetal medicine. There is minimal data regarding invasive management of fetal arrhythmia [1]. The majority of cases of fetal tachycardia are caused by supraventricular tachycardia (SVT). Multiple etiologies of fetal SVT exist, including reentrant tachycardia, atrial flutter, and ectopic atrial tachycardia [2]. Atrial tachycardia is characterized by a typical 2 : 1 ratio, with atrial rates up to 400 bpm and ventricular rates up to 220 bpm.

Different management options exist for fetal tachycardia; selection of management is dependent on gestational age, severity, associated congenital abnormalities, and maternal desires. The ultimate goal of treatment is to slow the fetal heart rate enough to allow adequate cardiac output. Progression to nonimmune hydrops fetalis (NIHF) is the most significant concern associated with fetal SVT. Significant morbidity and mortality rates (>50%) are seen in fetuses with hydrops, as evidenced in this case.

The development of fetal hydrops is considered an indication for transplacental therapy [3]. At term, a short attempt at medical management may proceed delivery planning. In the preterm period, treatment is warranted to avoid compounding the complications of prematurity with the morbidity of fetal hydrops [4]. This must also be balanced with maternal contraindications and risks of medication therapy.

Traditional management consists of transplacental treatment with antiarrhythmic medication [5]. Most centers, such as ours, begin therapy inpatient with daily electrocardiograms and cardiac monitoring for maternal well-being. With this treatment modality, 65-95% of fetuses without hydrops will undergo cardioversion within 2-7 days after initiation of treatment. Regardless of the medication selection, all show reduced efficiency in the setting of fetal hydrops [6]. This effect may be due to lower placental transfer of drugs as well as baseline cardiac decompensation.

Commonly used agents are digoxin, sotalol, and flecainide [7, 8]. Jaeggi et al. compared these three agents and concluded digoxin and Flecainide were superior to sotalol with respect to converting SVT to normal sinus [9]. Thus, historically, digoxin has been the initial drug of choice for with a reported success rate > 50%, yet these findings occur with levels greater than 2 ng/mL, which may have secondary maternal effects. A meta-analysis specifically investigating usage with fetal hydrops noted flecainide may be superior to digoxin under these circumstances [10].

Treatment is considered refractory if there is no improvement, or if there is deterioration of fetal status despite therapeutic levels. Under those circumstances, one must consider the gestational age and overall fetal survival prior to delivery planning. Postnatal treatment includes medical management as well as cardioversion [11].

An invasive approach allows direct and rapid steady-state levels in the fetus thus providing an alternative in cases of treatment refractory SVT [12]. Direct fetal therapy also circumvents the placental dysfunction which hinders therapy as previously described. Although few reports exist, most recommend continuing treatment with maternal digoxin to ensure the fetus remains cardioverted [13].

## 2. Case Report

**Figure 1:**
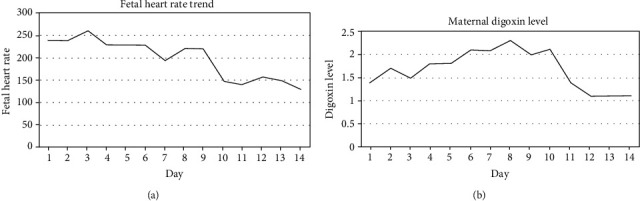
Fetal intramuscular injection of digoxin occurred on Day 9, and fetal heart rate normalized on Day 10 at 150 bpm (a). Maternal digoxin level. Day 1 represents maternal digoxin levels prior to the third dose of oral digoxin, dosed at eight-hour intervals. Levels returned to the therapeutic range on Day 11 (b).

**Figure 2:**
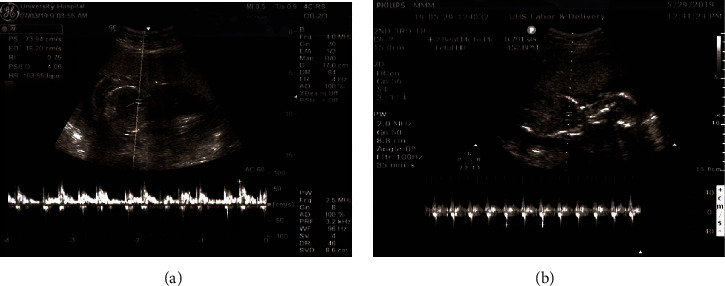
Transabdominal ultrasound of fetus prior to and after fetal intramuscular injection of digoxin. Fetal heart rate is 194 bpm after 6 days of treatment with oral digoxin (a). After the procedure, fetal heart rate is 152 bpm (b).

The patient was a 31-year-old G2 P0010 African American female with no significant medical history who presented with fetal tachyarrhythmia and nonimmune fetal hydrops at 24 weeks gestation. The fetus was noted to have a ventricular rate of 221 bpm and an atrial rate of 397 bpm. The ultrasound was also significant for fetal hydrops with ascites and scalp edema. Fetal echocardiogram revealed both pericardial effusion and bilateral pulmonary edema. Pediatric consultants recommended traditional treatment with oral digoxin 0.25 mg every 8 hours with maternal monitoring for digoxin toxicity. We continued oral digoxin therapy and monitored the fetal heart rate twice daily (Figure 1(a)). Maternal digoxin levels were monitored daily, with a goal of maintaining a level between 1.0 and 2.0 ng/mL, the level considered to be therapeutic for the fetus and risk minimal adverse effects to the patient (Figure 1(b)). This therapy was initiated, and the fetal heart rate was monitored yet remained greater than 190 bmp despite 7 days of treatment (Figure 2(a)).

After reviewing the available literature and discussing therapeutic options with the patient and weighing risks and benefits to both the patient and the pregnancy, we proceeded with fetal intramuscular digoxin injection. Some case series report of fetal intramuscular injection of digoxin, the only published series of invasive treatment of fetal tachyarrhythmia [12]. The recommended dosage of digoxin is 88 mcg/kg every 12-24 hours. In our case, 1 cc of 72.3 mcg of digoxin constituted in normal saline was injected with a 22G needle into the fetal buttock under ultrasound guidance, with dosing based on estimated fetal weight from the day of the procedure. Additionally, 10 cc of amniotic fluid was collected and sent for digoxin level (0.001 mcg). The patient tolerated the procedure without apparent complications. The fetal heart rate normalized within 24 hours of treatment (Figure 2(b)).

Although the fetal heart rate remained in normal sinus rhythm, at 30 weeks gestation, secondary to persistent scalp edema (and resolving abdominal ascites) decision was made to initiate steroid therapy for fetal lung maturity. At 31 weeks gestation, the patient developed preeclampsia with severe features. At this time, persistent fetal edema was thought to be a manifestation of mirror (Ballantyne's) syndrome. Fetal monitoring was nonreassuring at this time. Consistent with the patient's desire for intervention, the decision was made to proceed with a primary cesarean section. She delivered at 2,018 g male fetus with Apgar scores of 1 and 2. Umbilical cord pH was 7.04 with a base excess of 11, reflective of the fetal monitoring and fetal acidosis. Initially, the infant was intubated and paracentesis was performed to decrease abdominal distention and improve ventilation. Continued discussion with the patient about the infant's grim prognosis led to a change to comfort care. The infant passed shortly thereafter. Pathological examination of the placenta revealed diffuse villous edema with villous dysmaturity and multifocal intravillous hemorrhage. Our patient declined fetal testing or autopsy.

## 3. Discussion

Direct fetal therapy for SVT, in this case atrial flutter, has been documented as an intervention for gestations complicated by nonimmune hydrops [9]. Although multiple reports exist on varied regimens and outcomes for first-line therapy of fetal SVT, there is limited data on invasive approaches to treatment. Our data suggests that fetal intramuscular injection of digoxin can be successful for fetal cardioversion. Given how rapidly the steady-state level of digoxin is reached with intramuscular injection versus transplacental treatment, we recommend using this method in fetuses with hydrops and in cases that remain refractory to transplacental therapy after 48 hours of treatment.

mirror syndrome, also known as Ballantyne syndrome, is a rare complication of fetal hydrops in which the pregnant female exhibits fluid retention thus “mirroring” the fetus [14]. Mirror syndrome may also present with hypertension and proteinuria thus distinguishing this condition from preeclampsia may be difficult [15]. Mirror syndrome may also be considered a poor prognostic factor for fetal outcomes with reports of fetal death > 50% [16]. Treatment for mirror syndrome is resolution of fetal hydrops or delivery. In our case, despite returning to normal sinus rhythm, fetal hydrops did not resolve and the patient developed mirror syndrome. As no other identifiable cause of the hydrops was found, delivery was indicated for maternal benefit.

Although hydrops did not resolve, we believe that only with greater attention to this condition and willingness to further study outcomes with invasive treatment can we obtain data to better understand fetal response to therapy. This would also allow us to develop a standardized algorithm for management of these complicated and rare cases. In this particular case, the patient proceeded to develop mirror syndrome which required delivery for maternal health. It is likely that gestational age, severity of hydrops, and etiology of SVT play a significant role in response to therapy and resolution of hydrops fetalis. We recognize that our observations will require further studies to lead to a standardized approach for the treatment of fetal SVT.

## Data Availability

Data is restricted access due to patient privacy.
